# The role of primary cilia in myoblast proliferation and cell cycle regulation during myogenesis

**DOI:** 10.1247/csf.24067

**Published:** 2025-01-10

**Authors:** Zhichao Wu, Nuo Chen, Daisuke Takao

**Affiliations:** 1 College of Animal Sciences and Technology and College of Veterinary Medicine, Huazhong Agricultural University, 1 Shizishan St, Hongshan District, Wuhan, Hubei 430070, China; 2 Hubei Hongshan Labolatory, 1 Shizishan St, Hongshan District, Wuhan, Hubei 430070, China

**Keywords:** myogenesis, myoblast, proliferation, cilia, imaging

## Abstract

The process of mammalian myogenesis is fundamental to understanding muscle development and holds broad relevance across multiple fields, from developmental biology to regenerative medicine. This review highlights two key aspects: myoblast proliferation and the role of cilia in this process. Myoblasts, as muscle precursor cells, must undergo tightly regulated cycles of proliferation and differentiation to ensure proper muscle growth and function. Recent research has uncovered an essential role for primary cilia, hair-like sensory organelles on the cell surface, in modulating signaling pathways crucial to myogenesis. Cilium-mediated signaling appears to regulate various stages of myogenesis, including the control of myoblast differentiation. Furthermore, primary cilia undergo multiple cycles of formation and disassembly during myogenesis, presumably enabling detailed, context-dependent regulation of their functions. In particular, the regulation of myoblast proliferation through cell cycle control by primary cilia is an important topic that requires further investigation. By examining the interactions between primary cilia and myoblasts, this review aims to provide new insights into the molecular and cellular mechanisms driving muscle development, with potential applications for understanding muscle-related diseases and advancing therapeutic strategies. Additionally, advancements in imaging and image analysis technologies have become indispensable for studying these processes at the cellular level. This review also addresses these technological advancements and current challenges.

## Graphical Abstract

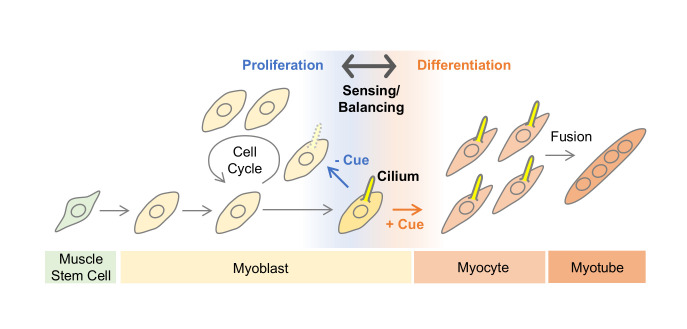

## Introduction

Muscles are essential tissues in animals, shaping the body and generating the force for movement. Understanding the process of mammalian myogenesis is therefore of great interest across multiple fields, including developmental biology, regenerative medicine, and animal husbandry. The outline of myogenesis is well known: 1) muscle stem cells (MuSCs) generate myoblasts through asymmetric cell division, 2) myoblasts proliferate, increasing in number and subsequently differentiating into myocytes, 3) myocytes fuse to form multinucleated myotubes, which then further mature into myofibers. While key differentiation marker genes expressed at each stage have been identified and much of the complexity in associated signaling pathways has been characterized, further insights are still needed (Gharaibeh *et al.*, 2012; Glass, 2003; Relaix *et al.*, 2021; Schmidt *et al.*, 2019; Zanou and Gailly, 2013).

In addition, the centrosome, which functions as a microtubule-organizing center and ensures bipolar spindle formation during mitosis, is involved in myogenesis: As myogenesis progresses, centrosomal proteins in muscle cells translocate to the nuclear envelope, transferring microtubule-organizing center functions to the nuclear envelope and leading to the loss of the centrosome (Becker *et al.*, 2020; Lucas and Cooper, 2023; Ng *et al.*, 2021).

While numerous signaling pathways and key factors have been identified, understanding the functional significance of primary cilia in myogenesis remains challenging. Primary cilia, composed of microtubules based on centrioles, which is the core of the centrosome, serve as cellular antennas to sense external cues in many important signaling pathways (Mill *et al.*, 2023). Primary cilia are involved in the myogenesis process, during which they undergo several rounds of formation and disassembly (Atmakuru and Dhawan, 2023; Ng *et al.*, 2021). Since mitosis requires proper disassembly of primary cilia to release centrioles and thereby form spindle poles, ciliary dynamics are closely tied to cell cycle regulation and cell proliferation.

Myoblast proliferation, which occurs early in myogenesis, is critical for proper muscle growth and regeneration, as muscle cells cannot increase in number through division once the irreversible differentiation process begins. However, unlike the well-explored early phase of MuSC activation and the later stages of differentiation and maturation, the intermediate stage of myoblast proliferation—and particularly the role of primary cilia during this stage—remains insufficiently understood. Therefore, this review summarizes current research on mammalian myogenesis, with a particular focus on myoblast proliferation and the role of primary cilia in this process. We also review technological advances and challenges in studying myoblast proliferation.

## Cilia Have Diverse Functions and Are Involved in Cell Proliferation and Differentiation

**Fig. 1:**
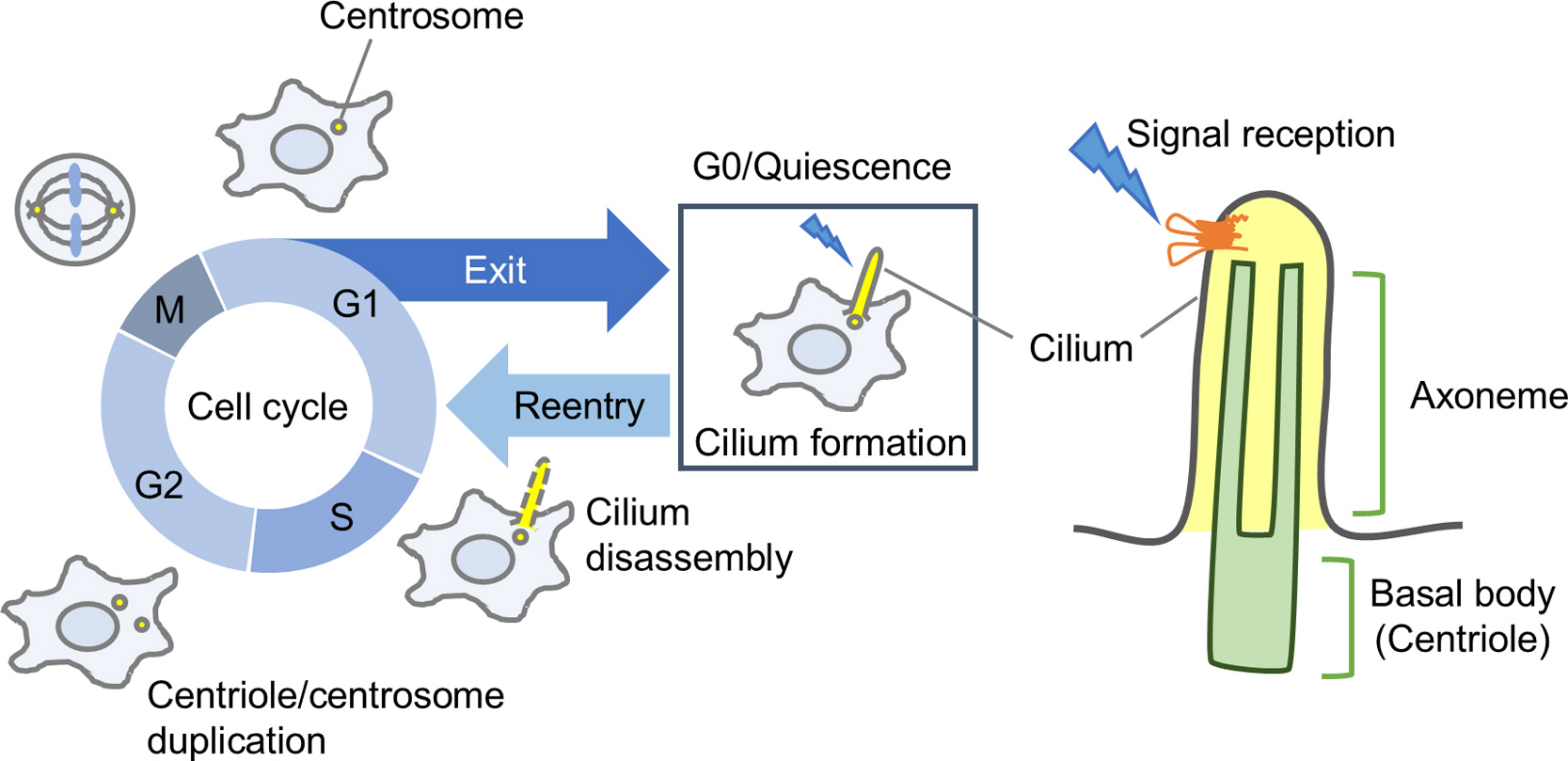
Cell cycle and cilia During the cell cycle, the centrosome duplicates in interphase, forming two centrosomes that serve as spindle poles in mitosis. When cells exit the cycle at the G1 phase and enter quiescence (G0 phase), microtubules in the centrioles, core structures of the centrosome, elongate to form cilia. These cilia, enriched with signaling receptors and cytosolic proteins, function as cellular antennas for receiving external signals. Upon cell cycle reentry, the cilia disassemble, and the centrosomes resume their roles as spindle poles. Cilia formation and disassembly are thus associated with cell cycle progression, suggesting a role for cilia in regulating cell proliferation.

Cilia are microtubule-based structures protruding from the cell membrane, which have long been recognized for their role in motility, such as propelling microorganisms and sperm or generating mucus flow in the tracheal epithelium. In addition to these motile cilia, non-motile cilia termed primary cilia (hereafter referred to simply as cilia) are present in almost all mammalian cells and function as cellular antennas that receive external signals (Mill *et al.*, 2023). When cells enter the quiescent state (G0), cilia are formed through the elongation of microtubules that comprise the centrioles, the core structures of the centrosome (Fig. 1). Although lacking the distinct membrane boundaries like those in other organelles, gating mechanisms at the base of cilia create isolated compartments with a unique composition of lipids, membrane proteins, and cytosolic proteins (Mercey *et al.*, 2024; Park and Leroux, 2022; Takao and Verhey, 2016a). Cilia are typically found in quiescent and differentiated cells, with their formation and disassembly tightly coupled to the cell cycle (Fig. 1). Through complex signaling networks, cilia play multifaceted, context-dependent roles in cell proliferation and differentiation by sensing external signals and regulating cell cycle progression.

As signaling centers that receive and transmit extracellular signals, cilia are pivotal in major pathways, including Hedgehog, Wnt, Insulin receptor (IR), insulin-like growth factor 1 receptor (IGF1R), and transforming growth factor-β (TGFβ)/bone morphogenetic protein (BMP) signaling. These pathways contribute to a wide range of biological processes, from embryonic development to postnatal tissue homeostasis (Anvarian *et al.*, 2019). Hedgehog signaling, one of the most thoroughly studied ciliary pathways, plays fundamental roles in regulating the proliferation and differentiation of adult stem cells and is involved in tissue homeostasis and cancer (Jing *et al.*, 2023). Cilia are also integral to muscle homeostasis and function, with specific signaling pathways discussed in detail later.

Upon re-entry into the cell cycle, primary cilia are disassembled. This disassembly is necessary for mitotic progression, as the basal body of the cilium serves as a microtubule-organizing center for spindle assembly. Proteins such as Aurora Kinase A (AurA) and Polo-like Kinase 1 (Plk1) facilitate this transition from G0 to G1 phase by promoting ciliary resorption (Goto *et al.*, 2013; Kasahara and Inagaki, 2021). Several pathways for ciliary disassembly, or resorption, have been identified, including partial or complete shedding of the cilium (Mirvis *et al.*, 2019; Phua *et al.*, 2017). While cilia disappear in cells that exit the quiescent state and reenter the cell cycle, the timing of this disassembly varies widely. Cilia may persist from late G2 to the onset of mitosis (Ford *et al.*, 2018), and it has been reported that cilia are even re-assembled in the G1 phase after an initial disassembly (Spalluto *et al.*, 2013). However, cilia have been shown to disappear in all cells by the onset of cytokinesis at the latest (Ford *et al.*, 2018), underscoring the importance of proper ciliary disassembly for cell cycle progression and thus cell proliferation. In addition to the loss of cilia, which is essential for cell cycle progression, the temporary retention of cilia may appropriately restrict external signal reception, allowing precise regulation of proliferation (Ho *et al.*, 2020).

## Cilium-Mediated Signaling in Myoblast Differentiation: Role in Cell Cycle Arrest and Induction of Myoblast Differentiation

**Fig. 2:**
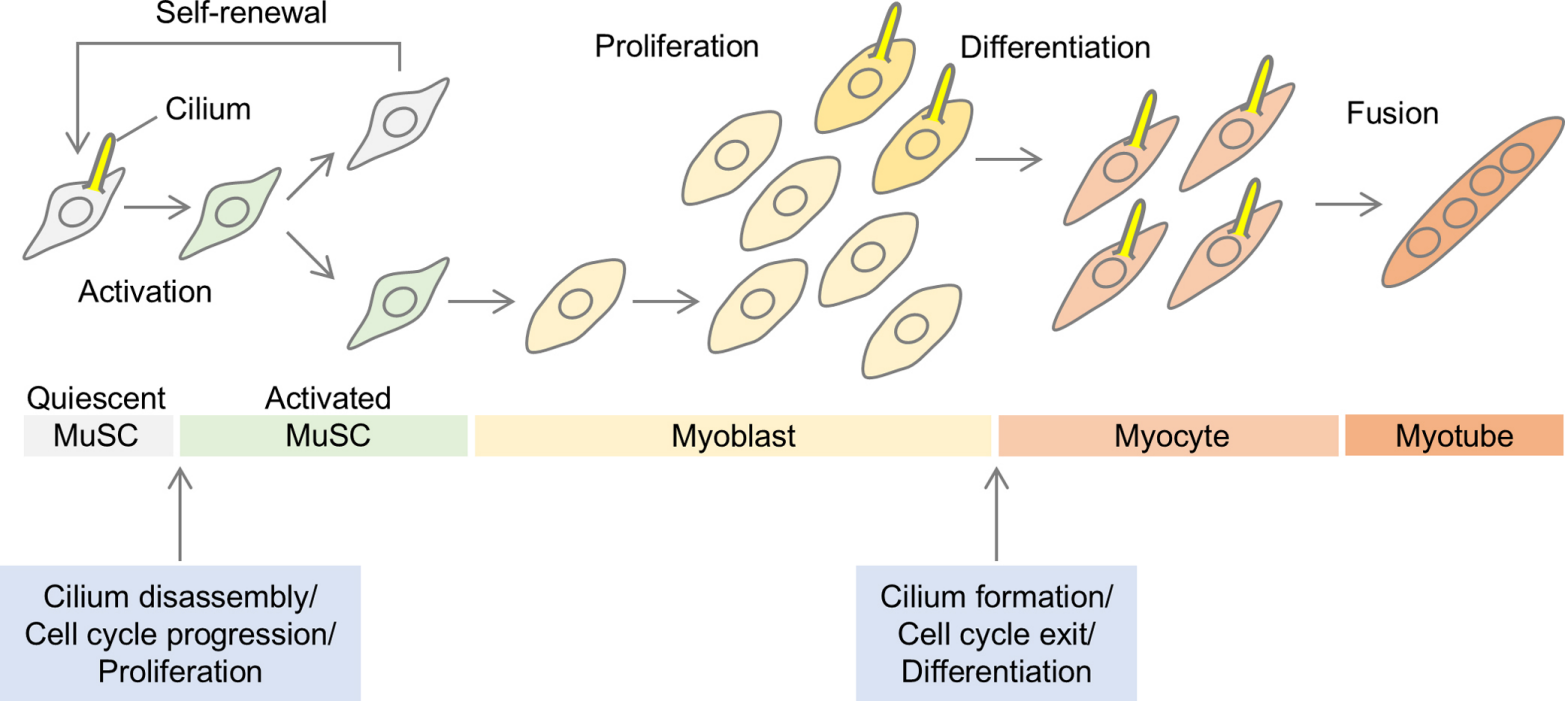
Myogenesis and cilia Activated muscle stem cells (MuSCs) divide asymmetrically to generate self-renewing cells and myoblasts committed to differentiation. Myoblasts increase in number during proliferation, and eventually differentiate into myocytes, which then fuse to form multinucleated myotubes and mature into myofibers. Quiescent cells, either pre-differentiation or during differentiation, can become ciliated, indicating that they may pause proliferation to receive external cues essential for advancing differentiation.

As outlined in several review articles (Byun *et al.*, 2024; Okino *et al.*, 2024; Yu *et al.*, 2021), myogenesis consists of a sequential differentiation process (Fig. 2). In developed muscles, muscle stem cells, also known as muscle satellite cells (both abbreviated as MuSCs), reside on the periphery of muscle fibers. MuSCs remain quiescent without proliferating but become activated in response to signals such as muscle injury, which triggers muscle cell regeneration. Once activated, MuSCs reenter the cell cycle and begin to proliferate, with asymmetric division leading to the self-renewal of some cells while others differentiate. Although further studies are needed to clarify the precise mechanisms, this fate decision of MuSCs is likely governed by a coordinated system of environmental cues and intrinsic factors (Fu *et al.*, 2022). In cells that commit to differentiation, the expression of the stem cell-specific Pax7 gene decreases, and they transition into myoblasts, which are characterized by high levels of MyoD expression and actively proliferate to increase in number. Myoblasts further differentiate into myocytes, marked by elevated levels of myogenin, and then fuse to form multinucleated myotubes, which eventually mature into myofibers. The expression of myosin heavy chain (MHC) increases around the time of myotube formation.

Interestingly, during this sequence of myogenesis, cells form and lose cilia, which correlates with the progression of the process and the differentiation state of the cells (Fig. 2) (Atmakuru and Dhawan, 2023; Ng *et al.*, 2021). Some MuSCs and myoblasts possess cilia, and their association with the cell cycle has been reported (Fu *et al.*, 2014; Jaafar Marican *et al.*, 2016). Cilia are assembled when cells exit the cell cycle and enter a quiescent state, a process crucial for establishing a non-proliferative condition. The cilium acts as a sensory organelle that dampens proliferative signaling pathways. The presence of cilia is linked to reduced signaling that promotes cell division, thereby maintaining myoblasts in a quiescent state (Venugopal *et al.*, 2020). It has been reported that during the asymmetric division of active MuSCs, self-renewing cells preferentially reassemble cilia (Jaafar Marican *et al.*, 2016), suggesting that the difference between MuSCs that arrest proliferation and myoblasts that continue proliferation may involve a mechanism mediated by ciliogenesis. In addition to cell cycle arrest and resumption, cilia—along with any remnants produced by incomplete degradation—likely play a role in the initial fate decision of MuSCs and the later transition of myoblasts from the proliferation phase to the differentiation phase (Ng *et al.*, 2021). While cilia play a key role in regulating differentiation, their involvement in controlling myoblast proliferation is a major focus of this review and will be discussed in further detail below.

## Myoblast Proliferation is a Complex Process Regulated by Diverse Molecular Pathways and Factors

**Fig. 3:**
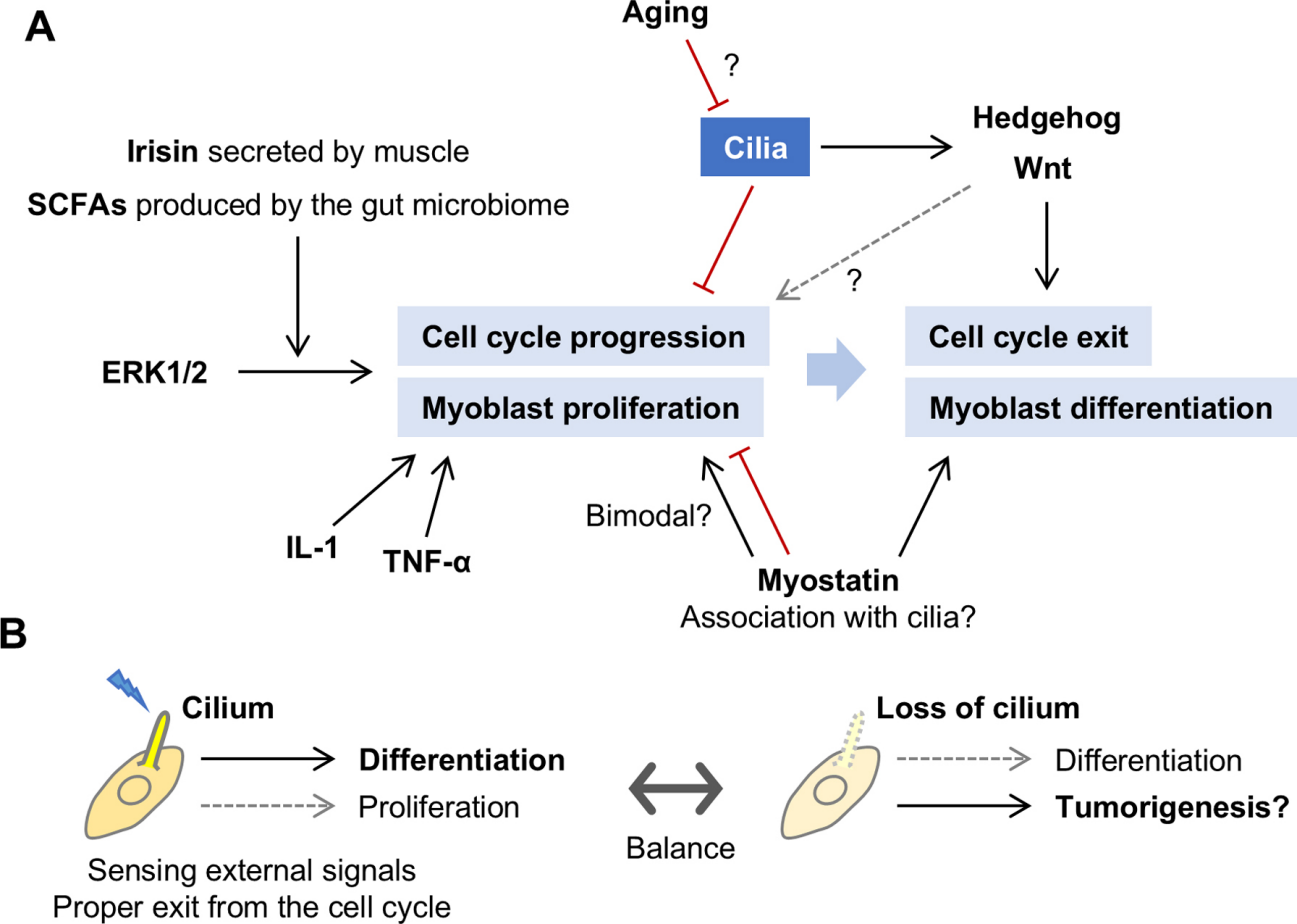
Pathways regulating the balance between myoblast proliferation and differentiation, and the role of cilia (A) Key pathways in myoblast proliferation and differentiation. Various external signals influence myoblast proliferation, its arrest, and subsequent differentiation. As some proliferative myoblasts are ciliated, cilia may act as sensors to monitor environmental conditions and thereby control the appropriate transition between proliferation and differentiation phases. (B) The role of cilia in guiding cellular decisions. Proliferative myoblasts occasionally form cilia, which may function as sensory modulators, interpreting environmental cues to guide the cells in either continuing proliferation or transitioning to differentiation based on the signals received.

The regulation of myoblast proliferation involves a complex network of signaling pathways, similar to many other cell types (Fig. 3A). One key pathway is the ERK1/2 signaling pathway, which plays a central role in maintaining myoblast proliferation (Feng *et al.*, 2013; Jones *et al.*, 2001). Activation of this pathway supports the cell divisions needed to accumulate sufficient myoblasts before differentiation occurs. When the ERK1/2 pathway is inhibited, myoblasts can undergo cell cycle arrest, which leads to differentiation and myotube formation (Feng *et al.*, 2013). In vitro studies have indicated that ERK-dependent myoblast proliferation is further promoted in vivo by endocrine factors, including irisin, a myokine secreted primarily by muscle (Lee *et al.*, 2019), and short-chain fatty acids (SCFAs) produced by the gut microbiome (Guan *et al.*, 2023). Research into communication and functional regulation between distant organs, including those mediated by the microbiome, is an expanding field, and myogenesis is no exception.

Myostatin, a member of the TGF-beta family, is a potent inhibitor of both myoblast proliferation and differentiation (Taylor *et al.*, 2001; Thomas *et al.*, 2000). Overexpression of myostatin has been shown to accumulate cells in the G2 phase, decreasing the frequency of cell cycle exit and thus inhibiting proliferation and differentiation (Joulia *et al.*, 2003). On the other hand, under certain conditions, myostatin has also been reported to promote myoblast proliferation (Rodgers *et al.*, 2014), suggesting a complex regulatory mechanism and potential limitations in using C2C12 myoblasts as an experimental model.

The activation of MuSCs and subsequent myoblast proliferation are essential components of muscle regeneration in response to injury. This regenerative process is regulated by a complex system that includes MuSC heterogeneity, diverse surrounding cells, and immune responses (Sousa-Victor *et al.*, 2022). In skeletal muscle regeneration, interleukin-1 (IL-1) has been shown to promote myoblast proliferation as a positive mediator within the inflammatory microenvironment (Chaweewannakorn *et al.*, 2018). Another pro-inflammatory cytokine, tumor necrosis factor-α (TNF-α), has been shown to promote early myoblast differentiation and satellite cell proliferation, suggesting that it may play roles at multiple stages of muscle regeneration (Palacios *et al.*, 2010). IL-1β and TNF-α promote myoblast proliferation through distinct pathways (Alvarez *et al.*, 2020).

Taken together, a wide variety of pathways, too diverse to cover comprehensively here, play roles in the regulation of myoblast proliferation. Given cell-cell interactions (Majchrzak *et al.*, 2024) and the diversity of gene expression profiles of MuSCs depending on their anatomical location and developmental origin (Yoshioka *et al.*, 2021), the regulation of myogenesis in vivo may be even more complex.

## Cilia as Key Regulators of Myoblast Proliferation

Cilia play a significant role in regulating myoblast proliferation and differentiation during skeletal muscle development. In addition to the general pathways mentioned earlier, recent studies have highlighted the critical functions of cilia in MuSCs and myoblasts, particularly in relation to various signaling pathways. Cilia are present on quiescent MuSCs but disassemble when these cells are activated and enter the cell cycle. This disassembly is essential for the transition from quiescence to proliferation, as the presence of cilia is associated with maintaining a non-proliferative state (Jaafar Marican *et al.*, 2016). Similar to other cell types, cilia seem to exert an inhibitory effect on myoblast proliferation (Fig. 3A). Indeed, disruption of cilium formation by knockdown of IFT88 in C2C12 myoblasts led to an increase in proliferative cells and a reduction in the expression of the quiescence marker p27 (Venugopal *et al.*, 2020). Once myoblasts lose their cilia and enter the proliferation phase, they can continue to increase in number until they regain cilia and proceed into an irreversible differentiation process.

The Hedgehog signaling pathway is involved in myogenesis (Fu *et al.*, 2014; Jing *et al.*, 2023) and has been reported to promote MuSC and myoblast proliferation and their differentiation into myotubes (Koleva *et al.*, 2005). Another important pathway that intersects with both cilia function and myogenesis is Wnt signaling (Liu *et al.*, 2022; Wheway *et al.*, 2018). The knockout of Talpid3, a gene essential for cilium formation, led to downregulation of Hedgehog and Wnt signaling and impaired muscle regeneration following MuSC injury, highlighting the importance of these signaling pathways for MuSC self-renewal (Martinez-Heredia *et al.*, 2023). Notably, pharmacological activation of Wnt signaling could restore regeneration, while Hedgehog pathway activation did not have the same effect (Martinez-Heredia *et al.*, 2023). While the complex signaling network, with its extensive crosstalk, is challenging to fully understand, the completely cilium-dependent nature of Hedgehog signaling—compared to the multifaceted Wnt signaling, which includes canonical, noncanonical, cilia-dependent, and cilia-independent pathways—highlights the indispensable role of cilia in regulating proliferation and differentiation in MuSCs and myoblasts. Indeed, in mice with impaired ciliogenesis in MuSCs due to IFT88 knockout, the muscle-strengthening normally induced by a Hedgehog agonist was abolished (Palla *et al.*, 2022). This interplay between cilium-dependent and cilium-independent pathways adds further complexity to the regulatory network of myoblast growth.

The regenerative capacity of muscle cells declines with age, partly due to decreased ciliation (Fig. 3A). Aged MuSCs exhibit reduced ciliary presence and Hedgehog signaling activity, but pharmacological stimulation of ciliary Hedgehog signaling has been shown to restore regenerative capacity in aged muscle (Palla *et al.*, 2022). Advanced glycation end-products (AGEs), which accumulate with age, cause muscle atrophy and contribute to sarcopenia (Granic *et al.*, 2023). AGE treatment has been shown to inhibit the proliferation of C2C12 myoblasts, coinciding with shortened cilia, although the cilia are not completely abolished in these conditions, and the functional mechanisms remain unclear (Suzuki *et al.*, 2023). Exercise-induced muscle hypertrophy is also impaired in mice with dysfunctional cilia (Li *et al.*, 2022), suggesting that cilia might mediate not only Hedgehog signaling but possibly mechanical sensing processes, with implications for the muscle atrophy and cilia loss seen with aging. It is well known that mechanical stimulation promotes myogenesis; for example, application of ultrasonic mechanical stimulation to pre-differentiated C2C12 myoblasts promotes differentiation and myotube alignment (Hashiguchi *et al.*, 2024). Further research is needed to clarify whether exercise-induced hypertrophy is mediated primarily by mechanical sensing via cilia or by chemical signals such as ciliary Hedgehog signaling. On the other hand, in some organs and cell types (e.g., kidney and pancreatic cells, as well as fibroblasts), cilia do not shorten or disappear with age; instead, they elongate, which can lead to dysfunction (Adametz *et al.*, 2023; Breslin *et al.*, 2014). Ageing has also been shown to reduce the regenerative response of MuSCs around the neuromuscular junction, the synapse between myofibers and motor neurons (Larouche *et al.*, 2021). Further studies are necessary to fully understand the relationship between aging and ciliary function in various contexts.

The role of cilia in the proliferation and differentiation of cells, including MuSCs and myotubes, during myogenesis is complex and sometimes appears paradoxical. However, focusing on the transition between proliferation and differentiation in myoblasts can provide a simplified framework for understanding this process. For example, it is reasonable to assume that proliferating myoblasts occasionally exit the cell cycle, utilize cilia to sense environmental cues, and then decide whether to resume proliferation or initiate differentiation based on the cues received (Fig. 3B). In this context, cilia may serve as a master regulator of this balance (Fig. 3B). Nevertheless, as mentioned above, the signaling pathways involving cilia form a highly complex network, requiring further research to unravel their details. Integrating analyses of intracellular microstructure dynamics with molecular biology, cell biology, and omics approaches could reveal changes in cellular profiles during the transition from proliferation to differentiation, which may shed light on the underlying mechanisms.

## Dysfunction of Cilia and Its Association with Muscle Diseases

Ciliary dysfunction is linked to a group of diseases collectively known as ciliopathies (Alzarka *et al.*, 2024). Since cilia play essential roles in regulating cell proliferation and differentiation during myogenesis, their dysfunction can contribute to the development of muscle cell tumors, such as rhabdomyosarcoma (RMS; Fig. 3B). Before the surge of research on primary cilia, Patched was identified as a gene implicated in RMS (Hahn *et al.*, 1998). Now known to encode a Hedgehog signaling receptor localized on cilia, Patched exemplifies the connection between ciliary signaling and muscle tumor development. Interestingly, in RMS cells, cilia may inhibit differentiation through abnormal Hedgehog signaling (Fu *et al.*, 2014). On the other hand, the Notch signaling pathway has been implicated in RMS (Roma *et al.*, 2011), indicating that non-ciliary mechanisms may also contribute to RMS development, although the interplay between these pathways remains under investigation. Beyond direct involvement in muscle cells, ciliary Hedgehog signaling within muscle-resident fibro/adipogenic progenitors (FAPs) also impacts muscle homeostasis, which may underlie the muscle-to-fat replacement observed in aging and dystrophic muscles (Kopinke *et al.*, 2017). Since aging-related MuSC dysfunction is partially due to reduced Hedgehog signaling activity within cilia (Palla *et al.*, 2022), a comprehensive analysis of the entire system, incorporating multiple cell types and age-related changes, may pave the way for new medical advances in muscle health.

## Technical Challenges in Analyzing Myoblast Proliferation and Differentiation: Accumulation of Ciliated Myoblasts

Studying cilia structure and function in cell culture requires effective methods for inducing ciliogenesis. Generally, serum starvation is a standard technique to arrest cells in the G0 phase and thereby promote ciliogenesis in cell models such as hTERT RPE-1 (human retinal pigment epithelial) and NIH3T3 (mouse fibroblast) cell lines (Moreno-Cruz *et al.*, 2023; Takao *et al.*, 2017; Takao and Verhey, 2016b). However, in myoblast cell lines such as C2C12, reduced serum not only induces quiescence but also triggers irreversible differentiation into myotubes (Andrés and Walsh, 1996), which complicates the enrichment of ciliated myoblasts.

To synchronize C2C12 myoblasts in the G0 phase, a unique approach involves suspending these cells in methylcellulose-containing medium (Gopinath *et al.*, 2007; Milasincic *et al.*, 1996; Sachidanandan *et al.*, 2002; Venugopal *et al.*, 2020). In this method, inhibition of the anchorage-dependent pathway allows myoblasts to be arrested in the G0 phase without undergoing differentiation. These arrested myoblasts can then re-enter the cell cycle when replated under normal culture conditions. Notably, ~20% of asynchronous, proliferative myoblasts exhibit ciliation, while suspension-arrested myoblasts show an increase to ~60% ciliation, which reverts to ~20% upon replating (Venugopal *et al.*, 2020). Additionally, cilia in G0-arrested myoblasts tend to be longer than those under normal growth conditions (Venugopal *et al.*, 2020), a feature associated with delayed cell cycle progression in other cell types (Kim *et al.*, 2011). Besides its methodological advantages, understanding the physiological significance of ciliary elongation in G0-arrested myoblasts may yield valuable insights.

Recent investigations into serum-free and alternative serum conditions underscore the complexity of serum’s effects on myoblast proliferation and differentiation. Particularly, as interest in cultured meat technology grows, serum-independent methods are being explored for their benefits in safety, cost, and quality control (McGillicuddy *et al.*, 2018; van der Valk *et al.*, 2010; Zhang *et al.*, 2020). For example, removing or replacing serum in C2C12 cultures alters differentiation properties, including myotube strength (Fujita *et al.*, 2010). New findings on how serum components influence myoblast physiology, such as metabolism (Jang *et al.*, 2022) and membrane cholesterol content (Katayama *et al.*, 2023), are beginning to emerge. Notably, serum deprivation does not affect the differentiation of chicken fibroblast-derived myoblasts obtained via transdifferentiation, indicating that a comprehensive understanding of the complex effects of serum components on myoblast proliferation and differentiation remains elusive (Ma *et al.*, 2024). Advances in this area promise insights that could foster both industrial applications and experimental setups optimal for studying myoblast cell cycle regulation and ciliary function.

## Technical Challenges in Analyzing Myoblast Proliferation and Differentiation: Extraction of Cellular Features from Images

**Fig. 4:**
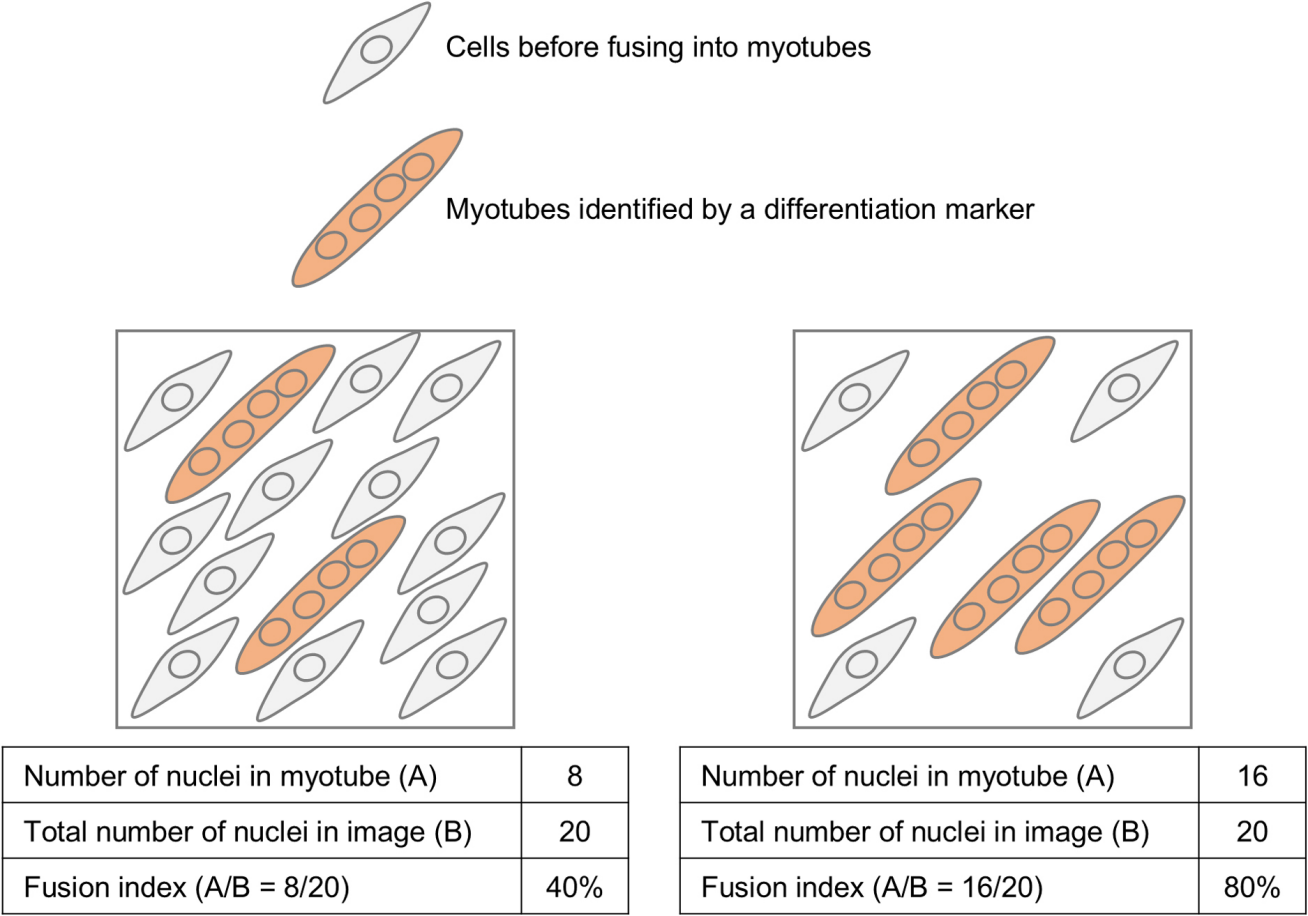
Image analysis of the myogenesis process The fusion index, a standard metric for myogenesis, quantifies the degree to which myocytes have fused into myotubes. It is calculated as the percentage of nuclei within myotubes in an immunofluorescence image stained for nuclei and myotube markers. In practice, myocytes and myotubes often appear fused or overlapping, presenting technical challenges in segmenting individual cells for single-cell phenotypic analysis.

Understanding cell biological processes requires detailed insights into the morphology and dynamics of cells and subcellular structures, with optical microscopy remaining pivotal in this regard (Karthikeyan and Asakura, 2024). Beyond conventional image analysis, artificial intelligence (AI)-based methods have the potential to uncover valuable, previously unexplored or inaccessible phenotypic features in cell images (Nagao *et al.*, 2020). In studying myogenesis, however, the complex morphology of myoblasts and myotubes poses challenges. Therefore, instead of analyzing the differentiation state of individual cells, researchers often use a common method to quantify the “fusion index,” which is the fraction of cells that have differentiated and fused into myotubes. This metric is determined by counting the number of nuclei located on myotubes out of the total nuclei in the image (Fig. 4). The use of fusion indexes has the advantage of being simple to compute even by hand, which can be further streamlined by using a deep learning-based tool (Weisrock *et al.*, 2024). In some cases, however, more detailed information about myotubes is required, such as their number, morphology, and biological activity. To meet these needs, specialized segmentation techniques for myotube analysis have been developed. For example, combining a watershed-based automated segmentation technique with a fluorescent biosensor has allowed researchers to observe variations in intracellular S6 kinase activity among myotubes (Inoue *et al.*, 2018). Similarly, the Myotube Analyzer software employs a comparable algorithm and offers a user-friendly GUI (Noë *et al.*, 2022). Another user-friendly myotube analysis tool, TRUEFAD (TRUE Fiber Atrophy Distinction), is available as a Fiji (Schindelin *et al.*, 2012) plugin that uses deep learning algorithms to measure myotube size and also supports analysis of histological sections (Brun *et al.*, 2024).

Single-cell analysis has become a mainstream approach for understanding cellular heterogeneity in biological processes, including myogenesis (Norris *et al.*, 2023). As gene expression analysis of individual cells yields novel insights, image-based methods for analyzing phenotypic profiles of individual cells have also gained importance. Unlike myotubes, which are multinucleated and uniquely shaped, general cell segmentation tools may be better suited for segmenting proliferating myoblasts. Numerous deep learning-based cell segmentation tools are now publicly available, including Usiigaci (Tsai *et al.*, 2019), a pioneering tool that uses the deep learning-based instance segmentation algorithm Mask R-CNN (He *et al.*, 2017). Among these, Cellpose has established itself as the de facto standard due to its high performance and accuracy, often eliminating the need for user retraining (Pachitariu and Stringer, 2022; Stringer *et al.*, 2021).

Although Cellpose is highly effective, further optimization may be necessary when segmenting myoblasts, as these cells exhibit relatively complex morphologies compared to epithelial cells with clearly defined boundaries. A notable issue in myoblast analysis is the heterogeneity within cell populations, which often contain cells at various differentiation stages, each with distinct morphologies. Instead of segmenting individual cells, researchers have developed a method to map differentiation states by dividing the image into small regions, each containing multiple cells, and classifying each region using a convolutional neural network (CNN) (Niioka *et al.*, 2018). While this strategy offers advantages for analyzing the myogenesis process, further refinement for individual cell-level analysis is awaited. Overcoming this technical challenge will be crucial for accurately analyzing the characteristics of individual myoblasts in images.

## Conclusions and Perspectives

Extensive research has provided substantial insight into the myogenic process. The morphological and histological dynamics of the process starting from MuSC activation, followed by cell proliferation and differentiation, and eventual cell fusion to form myotubes and further maturation, are now well characterized, alongside the complex molecular networks of signaling pathways that drive these events. A large body of work has highlighted the role of cilia, underscoring their universal importance in tissue and organ formation and function. In proliferating myoblasts, cilia appear to participate in signal reception and regulation of cell cycle progression, although further studies are needed to clarify these mechanisms. As omics technologies continue to standardize biological research, the development of advanced image analysis techniques for assessing individual cell functions has become increasingly crucial. In addition to conventional cell biological and physiological approaches, novel comprehensive techniques that integrate bioinformatics and bioimaging promise to accelerate muscle growth and regeneration research across both medical and agricultural sciences.

## Conflict of Interest

The authors declare no competing interests with the contents of this article.

## Author Contributions

DT conceived and supervised the review; ZW, NC, and DT wrote the manuscript.
